# Book Reviews

**Published:** 1815-09

**Authors:** 


### CRITICAL ANALYSIS

### OF RECENT PUBLICATIONS

IN THE

### DIFFERENT BRANCHES OF PHYSIC, SURGERY, AND MEDICAL PHILOSOPHY.

---

*An Essay on the Venereal Diseases which have been confounded with Syphilis, and the Symptoms which exclusively arise from that Poison: illustrated by Drawings of the Cutaneous Eruptions of true Syphilis, and the resembling Diseases.* By RICHARD CARMICHAEL, M.R.I.A. President of the Royal College of Surgeons in Ireland, and one of the Surgeons of the Lock Hospital in Dublin. 2d Part. 4to. pp. from 123 to 234.

IT is with high satisfaction that we announce the second part of this much-wished-for performance. 'Till the time of Mr. Hunter, the venereal disease was considered as nothing or every thing; for what has no description, and what appears in every form, are pretty much the same thing. It was not amiss that a poetical term should be applied to what it was found impossible to describe. Accordingly, the word *proteiform* was found particularly convenient, because, whilst it explained nothing, it silenced all inquiries. The laborious Astruc, who did the most to trace the history of syphilis, was, indeed, somewhat more accurate in his description of the true disease, but not less at a loss to account for the anomaly described by different writers, and especially by some who wrote earlier than the date to which he confined the origin of syphilis. At length, by the undefined use of the word cancer, he escaped the necessity of introducing Proteus. But the uncertain occurrence of secondary symptoms in different parts, and in a different form, was a difficulty he found it most convenient to solve by the introduction of another heathen deity. As this writer is now but little attended to, it may, perhaps, not be unentertaining to transcribe the passage in which he *poetically* explains himself. The poison, he tells us, may " retain a middle state, and be continually renewed as it flies off, but under such restraints as not to show itself. But, (adds he,) to this purpose, it is requisite that the poison which is admitted or left behind, should hold such measure in quantity and force, and the blood, likewise, keep such a temper in quality, and the manner of its generation, as to permit a renewal of the poison, poison, but such a renewal as is constantly one and the same, without addition or diminution. For otherwise, if the quality of the blood be vitiated by a fever or any adventitious disease, or errors in diet, immoderate watchfulness, drunkenness, &c. then, by the same means that the blood departs from its natural disposition, the before latent poison will gradually gain ground both in quantity and strength, be restored to its natural fierceness, and, like another PANDORA'S box opened, bring on a terrible group of grievous diseases, which will end in a manifest lues."*

Every reader of the present day will form the same opinion of the above passage, viz. that the author, not knowing what to say, availed himself of a language which no one could confute, because it was destitute of all meaning; and, to complete the whole, the allegory of Pandora is substituted for the fable of Proteus.

At length Mr. Hunter, with an accuracy which marks all his labours, undertook to ascertain the true diagnosis of a disease, in which he does not materially differ from his predecessors. Having thus established the exact and uniform character of the chancre, it was easy to see that whatever assumed a different form, must be a different disease. He taught us also the laws of the disease, by which we have been enabled to account for the uncertainty of its secondary appearance in a different form, and the circumstances under which we can assure our patient that he is free from all the effects of the poison, unless he exposes himself afresh.

Unfortunately, Mr. Hunter was not aware of the difficulties he had to encounter in rendering a new doctrine palatable, or even intelligible, or, if he was aware, he was unfortunate in his attempts to remove the difficulty. His book, consisting of almost entirely new matter, and rendered tedious by the unnecessary minuteness of the less important parts, was made still more complicated, by the number of hands said to be engaged in compiling it. Hence, though purchased with eagerness, it was little understood, till Dr. Adams published his "Morbid Poisons." This gentleman, availing himself of Mr. Hunter's discoveries, and having access to sources from which Mr. Hunter's want of early education excluded him, showed that all the ulcers found on the genitals, excepting the true chancre, and produced by many modern authors as forms of the venereal disease, are accurately described by Celsus, whose arrangement is so complete as to supersede the necessity of any other. Accord-

* Vol. i. p. 164. Barrowby.

 ingly, ingly, he follows the language and adopts the characters marked by the Roman physician, showing how exactly they tally with diseases which all but Mr. Hunter have confounded with syphilis.

Mr. Abernethy has since written several popular essays on the same subject, in a style so interesting, as very much to attract the notice of medical readers. But, if Dr. Adams was too philosophic in his mode of reasoning, Mr. Abernethy is not less diffuse. The great advantage, therefore, of his writings are, that they have brought the subject into more general inquiry, and excited doubts the solution of which must produce information and conviction. He, however, confounds all the appearances which are so accurately delineated by Celsus, under the common term of *pseudo-syphilis*, which is the more remarkable, as he does ample justice to the labours of Mr. Hunter, Celsus, and Dr. Adams. Mr. Pearson has availed himself of a somewhat similar expression, *cachexia syphiloidea.* His writings are, hovever, very serviceable in showing that there never was a period when, besides mercury, many other remedies, all of which he relates, were not considered for a time equal to the cure of chancres; that is, that other ulcers on these parts have always been confounded with the venereal, and hence other remedies have been supposed equal to the cure of a disease which more mature experience has uniformly assigned to mercury. Hence, he very reasonably concludes, that all the supposed venereal ulcers which had been cured by the acids, were a different disease. In this important inquiry Mr. Blair lent his assistance; but neither of them undertook to discriminate the venereal ulcer from those which might be mistaken for it.

About this time, Dr. Adams published his second edition of Morbid Poisons, which, we shall see, Mr. Carmichael has made the basis of his publication. It may be urged, then, that the latter was altogether unnecessary; but this was far from the case. Dr. Adams's work, though it will always be a standard for philosophical reasoning, requires study and leisure: like Dr. Willan's, it has found its way to every medical library, but, like his, is only referred to in order to solve difficulties. There was, therefore, great danger lest the important truths contained in both might be superseded by lighter performances. This danger became the greater, when a work, under a title similar to Willan's, had encouraged all that indolent scepticism which the long labours of Mr. Hunter were beginning to remove. In Dr. Bateman's "Synopsis," though the terms *Pandora's* box and *proteiform* disease are not actually adopted, yet the words only are omitted, omitted, the ambiguity remaining precisely the same, with this increased disadvantage, that the reader is taught to believe that, what cannot be described, may be distinguished by the eye. "The subject," says Dr. Bateman (of cutaneous eruptions the result of the venereal poison), "is indeed difficult, and *not yet sufficiently investigated;* for these eruptions assume such a variety of forms, that they bid defiance to arrangement according to their external character, and, in fact, they possess no common or exclusive marks by which their nature and origin are indicated."

The expectation of possessing all Dr. Willan's labours in a cheap form, was likely to give a considerable circulation to the above work, and has rendered such a performance as Mr. Carmichael's particularly necessary. By the advantage of a large hospital, devoted entirely to these diseases, he has been enabled to confirm, in cases out of number, all that was taught by Mr. Hunter. He has followed the arrangement of Dr. Adams, or rather the revived arrangement of Celsus. By these he has practically proved the accuracy of that much admired ancient, and shown, that what may be discriminated by the eye, may always be explained in perspicuous language.

We have dwelt thus long on the important subject of the work before us, because it is one on which so much has been written, and so little understood, till Mr. Hunter's time; and because, without perpetually reminding the rising generation of the labours of their predecessors, there is always danger of their falling into despair or indolence in the discrimination of diseases which are perpetually occurring. The specific remedy, too, is by no means a trifling one. When used judiciously to a well-ascertained venereal sore, its success is uniform. Other diseases are often exasperated by it, and the perpetual repetition of mercury in a mere empirical mode, or by guess, is inconsistent with the laws of regular practice, and often subversive of the patient's health.

We shall make one other remark, which an observation in one of our former numbers seems to demand from us. We mean the very imperfect manner in which this subject has been treated by the writers of the northern metropolis. Cullen, to whom we owe so much, consigns all he has to teach on this disease to a writer, who, for a time, acquired some celebrity from such a recommendation, but is now entirely overlooked. Mr. Bell, whose System of Surgery contained all the modern improvements of the art, has proved as deficient in this disease as he was respectable in his other labours. It is not, in our opinion, difficult to account for this: at least we strongly suspect that the *morale* of Edinburgh burgh is different, if not more severe, than of either London or Dublin. In size, Edinburgh bears no comparison with London; and in decency of manners, as well as freedom from mendicity, it is not less superior to Dublin. Is it for this reason that we hear of no Lock Hospital in Edinburgh, or of no writers among its medical officers? We hope these hints will not be lost on our brother critics. Highly as we respect their labours in general, they have been uniformly deficient in all their remarks concerning the facts which are gradually leading us to improvement in the diagnosis of syphilis, and its discrimination from other diseases.

This may appear a long preface, but it will not be found irrelevant; and, having thus cleared the ground, in resuming our notice of Mr. Carmichael's work, we need scarcely do more than offer a few extracts.

This "second part" opens with a chapter on the second class of primary diseases, which have been mistaken for syphilis.

"In this chapter, (says the author,) I purpose to enter on the consideration of the second class of primary diseases, which have been confounded with syphilis, viz. the phagedenic ulcer, and the sloughing ulcer, together with the constitutional symptoms arising from both species.

"The profession, as far as I am acquainted, is indebted to Dr. Adams, for distinctly pointing out the wide difference which exists between these ulcers and the true syphilitic chancre; and, in support of his opinion, he has extracted some passages from Celsus, which prove that they, or similar ulcers, were well known to the ancients.* But, notwithstanding his observations, and the testimony of Celsus, the great body of practitioners still continue to look on them as chancres, altered or modified by inflammation, or some morbid disposition in the constitution of the patient. I have ascertained a fact, however, which, I trust, will dissipate these doubts, and demonstrate that they arise from a poison totally distinct from the syphilitic, and obedient to other laws. This fact is, that the constitutional symptoms of the phagedenic and sloughing ulcers are totally different from those of chancre, and cannot, like them, be superseded by mercury.

"The ulcers which formed the subject of my third chapter were of a mild superficial nature; and, from the similarity of their constitutional symptoms, probably owe their origin to one common virus. Those, however, which I am about to consider, are violent and destructive; but seem to be also closely connected with each other, for the sloughing ulcer will occasionally assume the phagedenic, and the phagedenic ulcer the sloughing, appearance. The constitutional symptoms, also, of both species are much of the

" * Adams on Morbid Poisons, 2d Edition, p. 31."

same same character; so that it is not unlikely that they also arise from one and the same virus, yet that virus totally distinct from the subject of the preceding chapter.

"The phagedenic ulcer, as its name implies, has a corroding appearance, and neither exhibits granulations, or surrounding induration. It spreads sometimes with rapidity, causing the most destructive havoc in the course of a few days; and, unlike a chancre, instead of being checked by mercury, it is almost always rendered more inveterate and rapid in its progress by that mineral. It more frequently attacks the glans penis than any other part; but the ulcer usually proceeds to affect the prepuce, which it often entirely consumes, and, continuing its depredations on the corona and glans, at last effects their total destruction. When this event takes place, the ulceration usually receives a sudden and permanent check. At other times, a spontaneous hæmorrhage, owing to the destruction of the coats of an artery, occasions a favourable change. The hæmorrhage from this cause is often so profuse, that I have frequently found the patient's bed-clothes drenched in blood; and, in most instances, found it necessary to stop the hæmorrhage by ligature. It is an occurrence, however, that is in general fortunate to the patient, for in many cases the ulceration is stopped in its progress by this cause alone. More rarely it happens, that, notwithstanding every anodyne, and lenient application, the ulceration will gradually proceed, until the entire penis is destroyed. There is also another characteristic of this ulcer worthy of remark, viz. the frequent return of ulceration, after the part has healed, to the very same spot which was at first affected."

In reading this passage, it is impossible not to be struck with the candour of the author; yet he must excuse us if we require a greater attention to words. *Inveterate*, though often used in a figurative sense, yet, according to strict English, as well as true etymology, means nothing more than *grown old*, which cannot be the writer's intention here, or, if it is, such is obscurely expressed.

The following is the description of the *nigrities serpens* of Celsus, or sloughing phagedæna of Dr. Adams.

"The sloughing ulcer is still more untractable and destructive than the phagedenic; but the surgeon has seldom an opportunity of seeing its commencement, as the disease excites so little uneasiness at first, that, in general, its attack is unobserved even by the patient, until it has existed for several days. A small black spot, that resembles a grain of shot, in colour as well as in size, is its first appearance, which, if seen by the experienced eye of a surgeon, even at this early period, will at once be recognized as a slough or mortification extending to some depth below the surface. The slough will continue to increase sometimes to only three or four times its original extent, and at others till it engages a considerable portion of the penis before a line of separation can be observed served between the living and mortified parts. When the separation at length takes place, we do not find a clean granulating sore, as occurs in simple mortification; but a corroding phagedenic ulcer, which begins a new kind of depredation on the surrounding parts, equalling the virulence, but not the rapidity, of the sloughing process by which it was preceded.

"Suddenly those parts are attacked by severe pain, and afterwards assume a bluish cast, and on the following day they are found to be covered with a slough; and in this way this destructive malady continues to extend its ravages by alternate sloughing and ulceration, until in one sex the entire penis, scrotum, perineum, and pubes are destroyed; and, in the other, until the labia, nymphæ, vagina, anus, nates, and, I believe, even the bladder and uterus, are engaged in one extended and malignant putrefaction.

"But, if the ulcer is fortunately stopped in its progress, and a portion only of the penis is destroyed, the orifice of the urethra becomes so contracted in the new-formed cicatrix, that the urine passes with the greatest difficulty, unless the utmost care is taken to preserve the passage open during the healing of the ulcer. These circumstances are noticed by the accurate Celsus, who describes a certain blackness, which, though it does not excite pain, gradually spreads, and, if we do not resist it, makes its way to the bladder, when no assistance can be given. His manner of treating this ulcer is by the application of the actual cautery, if it is confined to the glans penis; but first he directs a small probe to be introduced into the urethra, in order to prevent that passage from closing. If, however, the ulcer has penetrated far, he recommends the excision of such parts as are engaged in the disease, and to be treated afterwards in the same manner as other gangrenes.

"It is unnecessary, after this description, to point out the difference between chancre and the sloughing ulcer. In fact, no two ulcers can be more unlike, not only in their appearance and progress, but in the manner in which they are affected by mercury. The former, an indolent ulcer, is immediately amended by its use; while, on the contrary, it only increases the malignity of the latter.

"The constitutional symptoms do not, as far as I have observed, differ in character from those of the phagedenic ulcer, as will be sufficiently apparent in the cases I shall hereafter detail. But they so seldom occur, that their infrequency deserves to be marked as one of the characters of the poison.

"As to the treatment of the sloughing ulcer, I must acknowledge that I am at a loss to lay down any general plan, as what I have found to be of service in one case has been of no benefit in another. However there are a few facts relative to its treatment which may be of advantage to practitioners to know.

1st. Mercury, as I have already hinted, should be carefully avoided, for even a mercurial atmosphere, or alterative doses of that medicine, will exasperate the disease.

"2d.

2d. Change of air, in every instance in which I tried it, was attended with decided advantage; and I am so fully assured of the benefit to be derived from this source, that the first thing I should recommend, on being consulted by a patient affected by a sloughing ulcer, would be his removal to another situation.

"3d. I have not observed any advantage to result from the use of emollient or fermenting poultices. When the sloughs are extensive, the constant application of a lotion composed of camphorated mixture and tincture of myrrh, combined in such proportion as will not excite pain, has been attended with much more benefit. It corrects the fœtor of the sloughs, and stimulates the sound parts to cast them off, but unfortunately it has not the power of preventing their renewal.

"4th. Bark appears to me to be injurious in this ulcer. I never observed any amendment under its use, but, on the contrary, found that the ulcer always rapidly extended during its exhibition. Indeed, from the high degree of fever present, we might, à priori, conclude that bark would not be serviceable. The pulse is, in general, from 100 to 130; and, when the ulcers are very extensive, the tongue is dry, brown, or even black, such as it appears in the advanced stage of typhus fever. A contrary mode of treatment seems to be indicated by the fact, that a spontaneous hæmorrhage from the ulcer frequently induces a favourable change.

"5th. The exhibition of opium or cicuta in large doses, has frequently been attended with the most decided good effects.

"6th. When the ulcer has gained considerable ground, we should entertain but a very unfavourable prognosis of the event. Thus, if the ulcer has already destroyed one half of the penis, the most judicious treatment will scarcely save the remainder, or prevent the scrotum from falling into mortification, in which case the patient (if he can think himself so) will be fortunate in escaping with life. But, if only a part of the prepuce or glans is engaged in the ulcer, however alarming the state of the patient, we may hope, under judicious management, to retrieve him from his perilous situation. I shall exemplify the circumstances I have advanced by the relation of three

*Cases of the rapid progress of the sloughing ulcer, which ended in the death of the patient.*

"Thomas Weldon, admitted March 18th, 1812. The entire penis was in a state of mortification; the glans had fallen off; the patient was in a very exhausted state; his pulse 120. He stated, that hæmorrhage had frequently occurred since the commencement of the complaint, which was upwards of six weeks previous to his admission. He had used mercurial frictions under the direction of a surgeon; and was at the time of his admission under the influence of mercury. All these circumstances sufficiently indicated that the further exhibition of that medicine would not be productive of any advantage.

"I ordered bark in powder, in such quantities as his stomach was capable of bearing; a pint of wine daily was also ordered, and a fermenting poultice was applied to the parts. I also directed a grain of opium to be given to him three times a-day, for the purpose of relieving irritation and pain, and in the hope that it might cause the same beneficial effects in this case, which it was known to produce in other instances of mortification.

"He derived considerable relief under this plan, for the pain was mitigated, and, in a few days, the sloughs began to separate, and on the 25th were entirely removed, leaving a clean phagedenic ulcer; but the entire penis, even to the pubis, was destroyed. His appetite and strength daily improved. He complained, however, of severe pain whenever he passed his urine, as it necessarily trickled on the ulcerated parts.

"On the 31st, at a time that he had made considerable progress towards recovery, he was seized with severe pain at night; and in the morning, the ulcer exhibited a blue and livid appearance, evidently evincing that another attack of the sloughing process was impending. The following day, the entire surface of the ulcer was in a state of slough. The process, in a few days, extended, with severe pain, over part of the integuments of the pubes, and down the anterior portion of the scrotum: his pulse was 120; tongue dry and brown. The bark, wine, and opium were repeated; and warm fomentations and fermenting poultices were applied to the parts.

"The sloughing process continued to extend slowly till the 16th of April, and then stopped, having destroyed a portion of the integuments of the pubes, and the interior part of the scrotum. The ulcer, after the separation of the sloughs, which left the testes completely uncovered, looked clean until the 2d of May, when the sloughing process recommenced on the posterior portion of the scrotum, which was soon destroyed. Considerable hæmorrhage took place during the night from the ulcer. The little powers of life he possessed became completely exhausted by this third attack. He, however, survived till the 9th of May."

Three other cases follow, described with equal minuteness, in which mercury was used, and all of which ended fatally. After this, the author relates several others which terminated favourably, remarking, with his accustomed candour, that at the time they came under his care, they were not nearly so far advanced as the foregoing. Two were unattended with any secondary symptoms. In the other two, secondary symptoms occurred, and were relieved by small alterative doses of mercury, though the primary sores were always exasperated by that remedy. This is perfectly consistent with the remarks Dr. Adams has made in his minutely-detailed case of sloughing phagedæna.

Some instances follow of constitutional affections, in which the author had not an opportunity of seeing the primary symptoms, symptoms, but which he judged proceeded from the phagedenic or sloughing ulcers, by their correspondence with others, which he had traced in all their stages. This chapter concludes with some very candid remarks on the first article of the last volume of Transactions published by the Medico-Chirurgical Society.

"It is with no small degree of satisfaction (says Mr. Carmichael) that I lately perused Mr. Ferguson's 'Observations on the Venereal Disease in Portugal, as affecting the British soldiery and natives;' as I conceive his statement of the progress of the symptoms, and the manner in which they were affected by mercury, together with the treatment found most applicable, strengthen the opinions I have offered on the nature of the phagedenic and sloughing ulcers. 'Tis true, he no where designates the primary venereal ulcers of Portugal by these terms; nor could it be expected that he should, as he evidently seems to be of the general opinion, that all venereal maladies spring from one common poison, the syphilitic. But I am inclined to believe, that the phagedenic and sloughing ulcers are far more general in Portugal than in these countries, and that their constitutional symptoms were actually the subject of Mr. Ferguson's observations. I ground my opinion on the following reasons, chiefly deduced from his own paper. The frequent melancholy mutilations which occurred among our soldiers, could only have arisen from the ravages of the phagedenic and sloughing ulcers, and not from the slow progress of a true syphilitic chancre, which would not probably have been checked by the mercury that was, in every instance of venereal infection, recurred to by the surgeons of our army. Besides, the name which the venereal disease in Portugal acquired among the British soldiery, *the black-pox*, reminds us of the most remarkable character of the sloughing ulcer. In the next place, the appearance of secondary symptoms, while the patient was strongly under the influence of mercury; and lastly, the manner in which the disease was treated by native practitioners, viz. by local applications, decoctions of the woods, and the exhibition of an insignificant quantity of mercury, when the disease attacked the bones.

"Mr. Furguson, after stating a variety of interesting particulars of the venereal disease of Portugal, which, one and all, reminded me of the symptoms and progress of the phagedenic and sloughing ulcers, comes to the following conclusion, which equally applies to those maladies as known in this country.

"1. 'That the disease, in its primary state, is curable in Portugal without mercury.

"2. 'That the antisyphilitic woods, combined with sudorifics, are an adequate remedy for constitutional symptoms, the quantity of mercury being always insignificant, and often altogether omitted; or,

"3. 'That the virulence of the disease has become so much mitigated by reason of general and inadequately resisted diffusion, or

 other other causes, that, after running a certain (commonly a mild) course through the respective orders of parts, according to the known laws of its progress, it exhausts itself, and ceases spontaneously.'

" As to his last deduction, we have to regret, that the constant interruption to the progress of the disease, by means of mercury, has, in these countries, prevented us from witnessing the gradual decline and exhaustion of the virus, as it seems is the case among the natives of Portugal; and there are strong grounds for believing, that the ravages which the venereal disease of that country committed on the British troops, arose, not so much from the inflammatory disposition manifested in the constitution of the inhabitants of colder climates on their arrival in a warmer, as from the indiscreet exhibition of mercury, a medicine from which, Mr. Ferguson informs us, the 'native practitioners religiously abstain, considering it with horror, as one of the poisons which foreigners madly wield.'

" Among several instances, which came under my observation, of the malignity of the venereal disease of Portugal, and the inadequacy of mercury for its removal, the following was the most remarkable:

" On the 9th of April, 1814, I was consulted by a young officer, who, three years before, had received a venereal complaint in Lisbon, which had produced the following catalogue of melancholy mutilations. The septum, alæ, and cartilage of his nose, had been destroyed by ulceration, leaving a wide extended opening into the nares. It had ceased to extend, and was nearly healed; but it still produced an offensive discharge. There was also an ulcer of the left leg, extending from the ham to the heel; the upper part was granulating, the lower foul and phagedenic: the calf of his leg had been entirely destroyed by the ulcer; and his leg was contracted into a permanent semiflex position.

" He stated, that the first symptom of his disorder was an ulcer, which, on examination, I found had destroyed the glans, and great part of the body of the penis. For this complaint, the surgeon of his regiment put him on a course of mercury, which, he stated, was of no service to his complaints; and that the ulcer extended rapidly, and by sloughs, under its influence—that, in a few months afterwards, an eruption of what he described as pustules appeared on his back—the ulcer of his leg followed, hard bumps rose on his cranium, and the bones of his nose became affected—that the ulcer of the penis healed, and the bumps on the cranium disappeared, under the use of nitrous acid—that he afterwards came to England, where he remained near two years, and underwent several severe courses of mercury by inunction. In short, that, from the commencement of his disease, to the period of his application to me, he was under the almost incessant action of that medicine. His gums were all destroyed by the repeated salivations he had undergone; and he was, at the time he applied to me, under its influence.

" A stronger

"A stronger instance than this of the inefficacy and injurious consequences of mercury cannot be adduced. I sent him to the country, where his family resided, with little else than directions respecting his diet, to abstain from mercury, and to take the compound decoction of sarsaparilla and antimonials. Since that time, I have heard that his health has considerably improved."

A chapter follows on tubercular eruption, not less interesting than either of the former; and which, we trust, may ultimately become much more so. The intractability of some of these cases by any remedy, induced Mr. C. to attempt the superseding of them by the inoculation of other morbid poisons. The safety and success of the plan was sufficient to authorise a continuance of the attempt, which the author's large opportunities will enable him to do, and which, we trust, he will not neglect, nor fail to communicate.

Mr. Carmichael now reverts to diseases resembling syphilis which arise spontaneously, or without contamination, from another subject. On this we would advise the author to assume his usual caution. We have seen in some females all the evidence of the secondary symptoms of lues, and of other morbid poisons, where we could trace no primary symptoms. We are ready to admit the great difficulty of trusting to such details; but it ought also to be recollected, that virulent gonorrhœa may appear in so slight a form as to be unnoticed, or mistaken for fluor albus. Even in men who have cohabited with none but unsuspected females, the same may occur.

Having thus gone through most of the appearances arising from, or mistaken for, syphilitic, and illustrated all with many satisfactory cases, Mr. C. concludes with a very useful epitome of the whole. An appendix is added, in which is related the further progress of some cases, the termination of which had not occurred when they were detailed in the body of the work.

Such are the contents of this truly valuable performance, a performance with which we are particularly pleased, as we trust, by the number of well-told and well-attested cases, it will produce the consummation of all that has been attempted by the sagacity of a Hunter, the close reasoning and learning of an Adams, the well-founded scepticism of an Abernethy, and the well-directed diligence of a Pearson. That it will teach the rising generation to attend to reason instead of dogma, shew them the necessity of industry instead of furnishing an apology for indolence, and, most of all, inculcate that modesty during their inquiries, which the difficulties acknowledged by such names ought to inspire.

*Observations*

*Observations on Pulmonary Consumption;* by HENRY HERBERT SOUTHEY, M.D. 8vo. pp. 174. Longman and Co. 1814.

As the subject of this volume is so common, and passes so frequently before the observation of every reader, it is scarcely to be expected that any new work upon it should possess much novelty, or contain much additional information. Authors, indeed, of a speculative turn will always produce something original, although they may follow a very beaten track; and, sometimes, improvements in practice have resulted from hypothetical opinions.

The work before us, however, is of a different complexion; it contains a large mass of facts selected from the most eminent authors, arranged perspicuously, and stated in more elegant language than is usually employed by medical writers. There is much matter condensed in a small space, and we have great pleasure in recommending the whole to the perusal of our readers, especially as our limits will only allow us to make a few extracts; but these will sufficiently attest the talents of Dr. Southey as a writer, establish his judgment as a physician, and prove that whilst he has freely availed himself of the observations of his predecessors; he is an original and coherent thinker.

In the first chapter we find a good practical distinction between strumous phthisis, and consumption arising from other causes. In all cases there is probably some shade of difference; but it is useful in practice to observe the division adopted by Dr. Southey, as the treatment in the two forms of the disease is different. From the second chapter we shall gratify our readers with the author's description of the appearances on dissection.

"On examining the lungs of those who have died of strumous phthisis, a number of vomicæ are usually found of different sizes; a whole lobe, and sometimes the entire lung of one side, will be found filled with them, small portions of lung in a sound state intervening. It is sometimes impossible to make an incision without cutting into an abscess. A substance is often found imbedded in the lungs of scrofulous patients, which in appearance resembles dirty wetted chalk, and feels like unctuous clay; but on exposure to heat neither melts nor inflames. The bronchial glands are frequently found much enlarged, and converted into a cheese-like substance. Mr. Carlisle describes the lungs of a consumptive patient in which the lymphatic glands at the root of the lungs were ulcerated, the substance of the lung being sound. Bony concretions are sometimes found in the branches of the bronchia, varying in size from a pin's head to that of a bean. These substances have been expectorated, and individuals have been known to discharge them them with a slight cough for many years.* When analysed they are found to consist of phosphat of lime. In that form of phthisis which is produced by inflammatory diseases, the substance of the lungs has been found nearly destroyed, and converted into a mass retaining barely a trace of its original structure, the portions of lung least affected adhering firmly to the investing membrane. Sometimes a large portion of the lungs resembles a sponge distended† with pus. I am not aware of the symptoms by which the existence of the large soft brown tubercle is to be suspected. Dr. Baillie describes it as a rare occurrence. Tubercles of a very large size will exist in the lungs without producing any considerable difficulty of breathing. In a patient who had been for some time under medical treatment for disordered intestinal functions, and whose breathing was affected only a few days before his death, a great number of very large peculiar tubercles were found in the lungs. They were firm, of a white colour, and nearly uniform in appearance throughout; some were entirely imbedded in the substance of the lung, while others appeared on the surface; they varied from the size of a fist to that of a nut. Nothing like suppuration appeared in any of them; but there was a slight blackish appearance in some, as if the substance of the lung had been imperfectly converted into the tumour. In the intervals of the tubercles the lung was healthy. The bronchial glands were considerably diseased.‡ A very small portion of lung in a sound state seems to suffice for mere existence.

"A man aged 60, who frequently spit blood during the course of twelve or fifteen years, died from hæmorrhage, after experiencing all the symptoms of pulmonary consumption, except the expectoration of pus. When the body was opened the lungs were found hard and shrivelled, so as to resemble half-burnt parchment. The inferior lobe of the right lung was the only part remaining sound, and of this lobe the edges were hardened. The mesentery was full of steatomatous concretions, and the omentum hard, and singularly contracted. The blood-vessels of the left side of the lungs

---

* Jam vero post longum sed fortasse non inutilem de his calculis sermonem illud constare, tandem vides quod hujus initio proposui, calculos inventos esse qui certe concreverint in bronchiis. In iisdem autem bronchiis, itemque in cellulis in quas ultimi ipsorum desinunt ramuli, plerosque concrevisse eorum quos memoravi, minimorum præsertim, credibile facit eorundem haud raro sine pure et sanguine secuta rejectio. Quod si crebri passim bronchiorum ramusculi materia quæ sic indurescere possit, obsideantur; intelligis qua ratione ab innata causa pulmones fiant lapidescentes et tophos lapideos ementientes aut materiam duram gypseam referentes.—Morgagni, lib. ii. epist. 15. c. 23.

† See Portal sur la Phthisie Pulmonaire, vol. i. p. 244. and Morgagni, epist. 22. lib. ii.

‡ See Lawrence in Medico-Chirurgical Trans. vol. iii. p. 77.

were were so nearly obliterated, that it was impossible to introduce into their principal branches the smallest pipe to inject them ; and those of the superior lobes of the right lung were so much contracted that their sides seemed glued together. The arteries which terminated in the inferior lobe of the same side of which the structure was yet sound were greatly dilated, a greater quantity of blood being forced there in consequence of the disease of the other lobes.* In a recent case which terminated fatally, the expectoration was never purulent, although all the other symptoms of the disease were well marked; cough, dyspnœa, hectic fever, emaciation, quick pulse, profuse expectoration of mucus. On examining the lungs, a number of small abscesses were found, not communicating with the bronchia."

The third chapter, which fortunately is a long one, contains numerous very interesting facts, and highly important remarks on the predisposing and exciting causes.

The treatment, which also occupies a space proportionate to its importance, forms the subject of the remaining chapters, from which we shall make some extracts. After some excellent remarks on the preventive means, the author proceeds to consider the remedies that are to be employed when the disease has actually commenced.

"Emetics have been strongly recommended in the early stages of pulmonary consumption. The sulphats of zinc and copper in particular are said to act like a charm; and certainly the immediate relief of dyspnœa, connected with fullness of the stomach, will follow their exhibition. From observing the utility of emetics in the tabes mesenterica of children, and their power of dispersing glandular tumours, it has been supposed that they may promote the absorption of tubercles in the lungs. I cannot say that I have ever seen permanent benefit derived from the use of emetics in this disease. In those cases which have been preceded or accompanied by hæmoptysis, the action of vomiting is always dangerous. When the expectoration is scanty and difficult, with a sense of oppression in the chest, and irregular febrile paroxysms, a dose of ipecacuanha sufficient to excite slight vomiting may be given with advantage.

"With regard to the different preparations of iron, I am convinced that in the great majority of consumptive cases they are decidedly injurious. In chlorotic females, of a consumptive family, this medicine as a preventive is invaluable, particularly when given in combination with some purgative. There is also a loud distressing dry cough, without fever or quickness of pulse, but causing loss of flesh and prostration of strength, which is removed by preparations of iron and nourishing diet. But, where purulent expectoration, quick pulse, and hectic fever, mark the disease, I

---

* See also Portal, vol. i. p. 35.

have never seen any advantage result from the use of oxyds or salts of iron; but, on the contrary, an aggravation of all the symptoms.

"Of all the medicines which have acquired reputation in phthisis pulmonalis, there is no one which varies more in its effects than digitalis, and none which is upon the whole more valuable. In some cases, when given even in very small doses, it reduces the frequency of the pulse, renders the expectoration easy, and alters the nature of the matter expectorated. In others it seems rather to increase than moderate the quickness of the circulation. Sometimes it produces intolerable nausea and vertigo, without in any degree alleviating the pulmonary symptoms, or seems to act only on the kidneys. In that form of pulmonary consumption which is preceded by hæmoptysis, of all internal remedies I consider it the most useful. In strumous constitutions it is less frequently beneficial; but no practitioner can have given it an extensive trial without being convinced of its occasional value."

"For the employment of opium in pulmonary consumption it is difficult to lay down any general rules. In the latter stages of the disease, when the alleviation of particular symptoms is all that medicine can effect, opium is invaluable; at an earlier period, when there is a probability of permanent recovery, it is oftener injurious than beneficial. If a few hours respite from the irritation of coughing is to be purchased at the price of increase of fever; if the sleep thus procured be disturbed by frightful dreams, and terminated by profuse perspiration and increased difficulty of breathing, it is evident that the progress of the disease is hastened rather than retarded by the remedy. I believe we may generally conclude, that when the sleep obtained by means of opium is attended with frightful dreams, it does more harm than good. Such sleep does not refresh, the mind is harrassed by imaginary evils, and the body more exhausted than it would have been by a sleepless night. There are cases in which none of these evils arise from the use of opium; and, although in general it increases the night sweats, in some instances I have known them prevented; and in others, that horrible anxiety by which they are frequently attended, obviated by a full dose of this drug. As it is impossible *a priori* to decide what individuals are favourably influenced by opium, the medical attendant can only be guided by the past experience of the patient, or the effect of a first dose."

We cannot suffer these observations on the effects of opium to pass without remarking that we have rarely found such unpleasant consequences from the judicious use of that valuable drug at any period of consumption; but we by no means advise its employment alone, having always combined it with other medicines, adapted to the particular case in which it was given

"Among the minor remedies which are employed to alleviate particular symptoms, the acid and neutral salts are to be noticed.

The sulphuric acid is valuable not only in hæmoptysis, and the form of phthisis which it induces, but whenever profuse perspirations are present is the best of all the refrigerants. Saline draughts are most useful when a dry feverish heat occurs in the early part of the night. When a patient lies awake, hot and dry and restless, a saline draught will sometimes procure sleep, where opium is either injurious or inefficient.

"From my own experience, I can say nothing in favour of the balsams. Are they of use in that form of consumption in which the purulent expectoration is only an altered secretion from the mucous membrane of the lungs? Blisters are often of essential service in the commencement of this disease, particularly when there is a general soreness and sense of heat in the chest. In a more advanced stage, when the object is to keep up external irritation, and when the pain is referred to a particular spot, a caustic issue is to be preferred. From the latter remedy I have seen very great advantage derived in many instances."

On change of climate, the author observes—

"The practice of sending consumptive patients to a warm climate has been long known, and is often attended with decided advantage. Such removals, however, generally take place too late. A variety of circumstances may render it impracticable for the invalid to migrate. To quit the comforts of home in search of health requires an effort, both in patients and their friends, which is rarely made till the progress of the disease renders it unavailing. Nor is a warm climate to be recommended in all forms of consumption: it is only strumous constitutions which are benefited by it. There is also considerable difficulty in choosing a place of residence which shall be free from the extremes of heat as well as cold. Should a consumptive patient remain in the south of Europe during the hot months, excessive warmth will accelerate the fatal termination of the disease; and to be perpetually flying either from heat or cold is inconvenient, expensive, and distressing. At Lisbon during the winter months it is often very cold, and few apartments are provided with grates or stoves. The Portuguese, as I have already stated, are in the habit of sending phthisical patients to Beja and St. Michael's. For such invalids the south or south-east coast of Spain offers the most desirable winter residence; and Valencia is the particular spot I should select. Hieres is to be preferred to any other part of the South of France. The consumptive should studiously avoid the vicinity of high mountains. Nice and Naples are therefore objectionable.

"Madeira has been much resorted to of late years; but the number who have recovered bear a very small proportion to those who have derived no benefit from their residence in that island. The accommodation for strangers is also extremely bad. Unless admission can be procured to the house of a resident merchant, it is sometimes impossible for an invalid to procure a decent lodging.

"The chance of the plague and the ophthalmia in Egypt, and of the yellow fever in the West Indies, are paramount objections to climates otherwise well adapted to the consumptive."

The hot-house system is next considered, and Dr. Southey cautions us not to rely upon it as a curative, but to expect the alleviation rather than the removal of consumptive symptoms from its employment. Of the tepid bath he speaks more favourably; and we are pleased with his remarks on the benefit accruing from regular exercise, both as a preventive and a remedy. The most preferable modes of gestation in consumption appear to be riding, sailing, and swinging. Riding on horseback, when the patient's strength is equal to it, we believe is very salutary; but, where circumstances will admit, a sea-voyage offers the fairest chance for recovery. For many other valuable observations on the effects of treatment and regimen in consumption, we must refer our readers to the volume itself, which deserves a place in every library.

---

*Edinburgh Medical and Surgical Journal*, No. XLIII. for July, 1815.

Art. I. and II.—*Description of an Annular Saw; with a Plate.* By T. Machell, Surgeon, Wolsingham.—*Description of a Circular Saw, and of a new Form of Bone Nippers.* By Charles Griffiths, Senior Surgeon to the Forces.

We are not fond of multiplying surgical instruments. When a man is master of his business, he is rarely at a loss for tools. The present, however, show an accurate knowledge of the subject, and are not without their advantage.—For a description of Mr. Machell's saw, see our last volume.

Art. III.—*Observations on the Fever prevalent in the Province of Guzzerat, with General Remarks on the Action of Mercury in the Diseases of India.* By A. Gibson, Bombay Medical Department.

No practitioner is ignorant of the importance of mercury in most complaints among the Europeans settled in India. But, after all that has been said, nothing is hitherto ascertained, excepting that mercury, by exciting a new action, or a disease which ceases with the causes, supersedes one which, if continued and increased beyond a certain degree, always proves fatal. We shall not, therefore, follow the reasoning contained in this paper concerning the "chronic inflammatory state of the lymphatic system;" nor the necessity of "the mercury being got in so as to dissolve this state in salivation." When the author informs us of the change he has found in the glands, by dissection after death, it will be time enough to reason about it. As little are we

 disposed disposed to inquire what name shall be given to the fever, whether typhöid or bilious; for, having seen something of East-India practice, we are not afraid to assert, that, whilst some men, retaining the College language, and associating with it certain College opinions, have been doubting about typhus, synochus, or synocha, the patient has been irretrieveably lost. If the symptoms run very high, it is often necessary to reduce them before mercury can produce its effect; and, frequently, the first reduction of the constitution would have cured the patient, without any exhibition of mercury. We are not saying this to lessen the value of that important medicine, but to hint to some of our Indian brethren the necessity of attending more to symptoms, and less to names. Happily, the manner in which the professional duties are regulated in India, imposes on every tyro the necessity of seeing practice before he engages in it. Let him be careful to improve this noviciate season, and also to consider himself as a learner all his life-time. Let him, therefore, tell us what he sees; but say nothing of typhöid, without telling us what it means; nor of bilious, till he has examined the livers of the diseased; nor of glands, till he has had the same opportunities in those organs also.

Art. IV.—*Observations on the Nature of the Climate, and the Fevers which prevail at Seringapatam.* By A. Nicoll, M. D. Member of the Royal Physical Society of Edinburgh, &c.

This paper is written by a gentleman of some observation; but, for the reasons mentioned above, we think very few practical advantages can be gained by such fugitive pieces. Those who embark in the Indian service have the best of all means of acquiring knowledge; and to others, the peculiar locality and consequent diseases are only interesting as registers. Again, however, we enter our protest against this affected nomenclature, which can do no good, and may be attended with infinite danger. What is typhoid, and what is the typhus icterodes of Cullen, but names? The author speaks of the latter as a *species* with as much ease as if fevers, like plants, could maintain precise characters, by which they are always to be distinguished. We copy the following paragraph, just to show to how little purpose the best practical men may reason concerning causes.

"With regard to the infectious nature of the yellow-fever, some doubts are entertained, from never observing a single orderly attending those ill with the disease, or any of the other patients in hospital, who were oftentimes indiscriminately mixed together, for the want of room to put our sick and convalescents in, contracting the disease. However, the prevalence of this disease being regulated lated in its operation by a determined range of atmospheric heat, and, from numerous facts related, especially by that enlightened physician, Sir Gilbert Blane, I have no doubts but that, under certain circumstances, in regard to the constitution of the atmosphere, and the susceptibility of individuals, it may evince an infectious nature. It is certain, that *marsh* and *animal* effluvia are never found to act but when they are near to the sources from whence they issue; and it is probable, that the difference of their effects may always be traced to these sources, as modified by internal causes, and peculiar constitutions of the persons affected. We are still ignorant of the chemical nature and exact sources of human and marsh effluvia; but it appears they are sometimes intimately combined, and, according to the state of combination in which they exist, the different varieties, seemingly, of contagious fever are produced. The various remote causes of fever have not been well ascertained, but the genera and species of fever have been pretty clearly pointed out; and, in each of those species, it is probable the effluvia are of one specific nature, though arising from one common source, but acquiring their peculiar characters from certain states of the atmosphere. The yellow-fever may, therefore, only arise from a combination of *animal* and *marsh* effluvia, operating under circumstances as above pointed out."

We do not know that Sir Gilbert ever practised in the East Indies. An *infectious nature* must mean *infection* or nothing. What are these *contagious* fevers which appear under such varieties; and what are genera and species; and, lastly, what are the "circumstances above *pointed out*"?

After making thus free with Dr. Nicoll's reasoning, it is but fair to add, that his account is enriched by examinations after death, and by practical remarks, which evince diligence and good sense.

But a single quotation he gives from Dr. Jackson is worth volumes of all the reasoning which can be produced about typhoid symptoms and typhus icterodes:—"When the disease has just commenced in any constitution, whether robust or plethoric, or weak and emaciated, if there are symptoms of any inflammatory diathesis, bleeding must be employed."

Art. V.—*Experiment, tending to prove that Gonorrhœa, and a smooth superficial Ulcer, without Induration, arise from one and the same Virus.* By SIMON M'COY, Surgeon, Dublin.

This is a pretty little experiment, valuable, inasmuch as the whole history is well-attested, having occurred in the person of the narrator. We shall transcribe it, with a few remarks which have not occurred to him.

"I beg leave to submit to your notice the result of an experiment I made upon myself about six months ago, at which time I mentioned mentioned it to Mr. Carmichael, as an interesting case, tending in the strongest manner to support the doctrine he promulgated, that gonorrhœa, and a smooth superficial ulcer, without induration, arise from one and the same virus. I have delayed the publication of the experiment, as I wished to ascertain if secondary symptoms would occur; and I have the *comfort* to observe that, as yet, nothing of the kind has appeared. I am aware that the experiment has been made before, to prove whether the matter of gonorrhœa can produce a disease incurable without mercury; but the fact being established that it will not, little attention appeared to have been given by the experimentalist to describe the sore produced by this virus. To learn the appearances which such a sore would be likely to put on, I made the following experiment.

"On the 14th of August, 1814, I procured the infection from a man who had a severe gonorrhœa, and applied it, on the end of a blunt silver probe, to the inside of the urethra, about two or three lines from the orifice. I next infected a lancet, and drew the edge two or three times over the prepuce, near the *corona glandis.* The application drew no blood, and in this state I allowed every thing to remain. On the morning of the 18th, I perceived the discharge had commenced from the urethra, and the part of the prepuce which had been inoculated felt very sore. I laid a little dry lint on it, and suffered the disease to proceed, without taking any medicines, or even using any precautions as to diet, until the 22d, when the discharge from the urethra was completely established, and one sore had formed on the prepuce, elevated in the centre, of a glassy shining appearance, and of a dusky yellow colour. It appeared not very unlike, in form, the section of a pea, and about this ○ size. Its boundary was accurately marked by a capillary circular line, of a bright red colour. This was made very evident by a magnifying glass, and the ulcer, through the same medium, appeared as if covered with grains of very fine sand. As my sole motive for infecting the urethra was to observe if it produced symptoms exactly the same as gonorrhœa taken in the usual manner, and as the point was settled in the affirmative, I entered upon the cure of that part of the disease, by injections of calomel and mucilage of starch. I had felt much inconvenience from *ardor urinæ;* but it began rapidly to diminish, as did the discharge, and in something more than three weeks it was entirely gone. I suffered the ulcer to go on, without any application but dry lint, and I took ten grains of Rufus' pill. This was all the medicines I took until the 29th, when the sore was entirely healed. I thought I was now done with an affair which had given me considerable trouble and uneasiness; but, on the 2d of September, I was astonished in the morning to perceive three sores make their appearance. Two were on the prepuce, near the seat of the former one, and one on the *glans penis;* the last was rather superficial, but the two former were accurately excavated ulcers, with elevated edges, but without the slightest induration. Their bases were were inflamed; the colour of the ulcer was the same as the primary one, if I may so call it. I allowed them to take their own course until the 8th, when, seeing no material change either in their character or appearance, I began to dress them simply with calamine cerate, and took every morning a pill of four grains of Rufus' mass. The ulcers began to decline rapidly, and by the 24th or 25th they were entirely healed.

"I have now detailed, as well as I am able, the circumstances of this experiment. Mr. Gregory, one of the pupils of the Lock Hospital, witnessed it in all its stages, and can corroborate my statements. I feel happy in presenting it to the public, as it so decidedly confirms the opinions of the gentleman above alluded to, who has so happily elucidated a disease which before was very imperfectly, if at all, understood."

On this little history, we would first remark, that the whole experiment leads only to a single fact, which has been long since admitted, namely, that gonorrhœa, as well as coriza or catarrh, may arise from a variety of causes, and of course from one which, on a less secreting surface, may produce an ulcer. But, if our author conceives this a sufficient proof that gonorrhœa is never venereal, or, as Mr. Carmichael would term it, syphilitic, that is, that it never arises from the same poison as on the prepuce would produce the venereal chancre, he will find himself mistaken. Dr. Adams has shown that the variolous poison on a secreting surface will produce only an increased and altered discharge, which discharge, when inoculated on a non-secreting surface, will produce precisely the same effect as the matter from a small-pox pustule. Mr. Hunter has also proved, by an experiment similar to Mr. M'Coy's, that the matter of gonorrhœa will induce chancre by inoculation. Nothing in this contradicts Mr. M'Coy's conclusions, but it shows that the variety of sores in these parts is as numerous as was pointed out by Celsus,* with the addition of a new poison which has arisen since his days, and which, as Dr. Adams remarks, is the only form of disease in the genitals, described by modern authors, which was not noticed by that accurate writer.

Art. VI.—*Case of obstinate Gleet cured by the Use of Sea-water as an Injection.* By Edward B. Fletcher, Surgeon.

A confirmation of the remark of Mr. Hunter and his commentator, that, when gleets become chronic, or, as Mr. Fletcher expresses himself, obstinate, it is right to try some new stimulus which may supersede the former long-continued ac-

* See his chapter De Obscænarum Partium vitiis.

 tion.

tion. Sea-water seems a very desirable formula, and we do not recollect that it has ever been tried before.

Art. VII.—*Case of Tænia cured by Spiritus Terebinthinæ.* By ROBERT HARTLE, Surgeon of the Forces, &c.

In this instance, two ounces of the above remedy were given fasting, without permanent injury.

Art. VIII.—*On Oil of Turpentine in Epilepsy.* By DAVID LITHGOW, M.D. Coleraine, Ireland.

This paper is written with much candour, containing an unsuccessful as well as a successful case.

Art. IX.—*Case of temporary Loss of Voice from Hysteria, occurring in a Recruit in the Honourable East India Company's Service.* By J. A. D. WATSON, Member of the Royal College of Surgeons, London.

Art. X.—*Case of Aphonia occurring during the Eruptive Stage in Measles; and two Cases of Contraction of the Fore-arm and Hand, removed by the Use of a Splint and Bandage.* By EDWARD SCUDAMORE, Member of the Royal College of Surgeons, London.

These papers contain good practical hints, which their titles sufficiently explain.

Art. XI.—*Case of Tetanus, in consequence of a Wound successfully Treated.* By Dr. REID, Kilmarnock.

Mercury was given very early, and applied to the wound; opium very early, and in very large doses.

Art. XII.—*Case of Convulsions after Delivery.* By THOMAS DUNCAN, Surgeon, Grenada.

Art. XIII.—*Case of Labour, with Apoplexy, Convulsions, and Flooding.* By G. F. EDWARDS, Member of the Royal College of Surgeons.

Art. XIV.—*Case of Re-union of the first Phalanx of the middle Finger, in a Letter to Dr. William Balfour, Edinburgh.* By HENRY W. BAILEY, Esq. Surgeon, Thetford, Norfolk.

This case is more remarkable than Dr. Balfour's, inasmuch as the subject was older.

" A labouring man had been supplying a chaff-machine worked by a horse, and entangled his middle finger with the knives, by which the first phalanx was completely cut through at the middle of the bone, and had been separated nearly an hour and a half before I saw it. The joint was not in the least injured. I was willing to try how far re-union of the parts might be effected; and, having cleansed the parts, I re-applied them carefully together, and secured them by plasters and small pieces of cards by way of splints. I desired the man to call upon me in a week's time, fully expecting to find the end mortified. To my astonishment, re-union had taken place, pulsation was felt distinctly at the end of the finger, and its colour was healthy; the nail separated at the fortnight's end; he complained of numbness in the finger. I have pleasure in saying, the re-union was completely effected in about five weeks from the accident. The wound healed by the first intention, and only required three or four dressings. I saw the man yesterday: he felt no pain in the finger. It is as strong as the others, and the bone is perfectly united, but he has not the power of bending it with the other fingers. The nail, I understand, had been bruised some time before the accident, and probably would have separated had not he met with the subsequent occurrence."

Art. XV.—*Observations on the Necessity and Utility of Blood-letting in Continued Fever, more especially in the Ardent Fever of the West Indies, commonly called the Yellow Fever.* By John Allen, Surgeon, R. N.

This is a very long and good practical paper. But we need only transcribe the following passage to show how many lives might have been saved, if the deserving Dr. Jackson had been attended to with decency, not to say with that respect to which his courage, arising entirely from the strength of his judgment and the accuracy of his observations, entitled him.

"As the object of communicating these cases is simply to exhibit the admirable effect of blood-letting in continued fever, so as to draw the attention of others to the subject, and to prove the indispensable necessity of adopting the practice, especially in the yellow-fever, I do not, in these observations, attempt to inquire particularly into the advantages to be likewise obtained from other means, being quite convinced, that, without blood-letting, no other remedy, however beneficial, when used as an auxiliary, can with safety be depended on. Before detailing any of the cases in which I have observed the great advantage of blood-letting, I may state, that, when I found the opinions favourable to this practice openly professed, but not publicly condemned,—boldly and sincerely asserted, but not attempted to be denied or refuted,—while, on the other side, it was admitted that blood-letting might occasionally be advantageous, I resolved to resort to experience as my guide, to be led to a decision only by the most positive facts, and therefore to pay particular attention to blood-letting in any cases of fever I might meet with. My services, till last year, being confined to ships in the English Channel, I have had but few opportunities of making observations of this kind in Europe. Indeed, I cannot say that I have since seen any case of continued fever, and therefore I do not pretend to assert from experience that the practice recommended is to be extended, without some caution, to fevers arising from contagion. Judging, however, from analogy, from what I recollect having seen of such cases in the more early part of my professional life, and from the authority of the celebrated Sydenham, I cannot doubt that blood-letting, the effects of which are so very different from what those educated in the doctrines now in fashion would expect, will at last be found to be consistent with nature, and to be the best and most successful method of treating all fevers, even the plague itself. The following case, which occurred at Portsmouth, served, with some others, to confirm in my mind the impression in favour of these opinions, and to impair the confidence I at first had placed in the mode of treating continued fever usually pursued in Britain.

" 'John Hogg, seaman, 21 years of age, of a slender habit, was attacked, on the 20th May, 1811, with shivering, sickness, vomiting, head-ach, and general uneasiness. His bowels were that day purged with senna; he was bled to the extent of 14 ounces, and in the evening he took an emetic. During the night he perspired freely, and felt himself, the following morning, almost free from complaint. In the course of the day, however, the head-ach returned; and, in the evening, had increased to a violent degree, being attended with a sense of cold, violent and sudden starting, and derangement of the mental faculties. I found him tossing about in bed, with a great tendency to vomiting, complaining very much of his head, neck, and eyes. His skin was extremely hot, though he complained of cold; his pulse was slow and firm; his lips and tongue parched and furred; and he took drink very greedily when offered to him, though he said he felt no thirst.

" 'Twenty ounces of blood was taken from his arm, and then he took an emetic, which brought up a quantity of crude ingesta. About an hour afterwards, the delirium appeared to be returning, and 25 ounces of blood were taken from another vein. It was of a florid colour, and came jet by jet from the vein, though situated at the radial side of the arm, and on that account was not liable to be affected by the pulsation of the artery. Towards the end of the operation he opened his eyes, and expressed himself relieved. A large blister was then applied betwixt his shoulders, and he soon fell into a very calm sleep, and was covered with a gentle perspiration.

" 'Early next morning, although he declared himself much better, and was free from any appearance of delirium, I thought it necessary, on account of the state of his pulse and of his eyes, to take 30 ounces of blood from his arm, which had a very tough firm coat, of a pale blue colour.

" 'At noon he was conveyed to Haslar Hospital, complaining of nothing except debility, which, however, was not so great as to prevent his walking without any assistance from the boat to his ward.

" 'He returned to the ship after a few weeks perfectly cured.' "

Yet for a similar practice, though successful, Dr. Jackson was calumniated, degraded, and forced to the only defence which remained, and which ended in six months incarceration. It is some relief to reflect that he has since arrived at the rank, which had he attained sooner, might have preserved the lives of thousands. Instead of any further remarks on this paper, we shall only recommend a careful perusal of his writings.

---

### MEDICAL AND PHILOSOPHICAL INTELLIGENCE.

